# Restoring expression of Stathmin-2: a novel strategy to treat TDP-43 proteinopathies

**DOI:** 10.1038/s41392-023-01533-1

**Published:** 2023-07-12

**Authors:** Sonja Menge, Lorena Decker, Axel Freischmidt

**Affiliations:** Department of Neurology, Ulm University, 89081 Ulm, Germany

**Keywords:** Diseases of the nervous system, Target validation, Neurological disorders, Molecular medicine

In a recent study published in *Science*, Baughn et al. revealed that TDP-43 acts as a steric block in *STMN2* pre-mRNA processing preventing inclusion of a deleterious cryptic exon, and present strategies for substituting this function to compensate for the loss of nuclear TDP-43 in a variety of neurodegenerative diseases (NDs) including amyotrophic lateral sclerosis (ALS).^1^

**Fig. 1:**
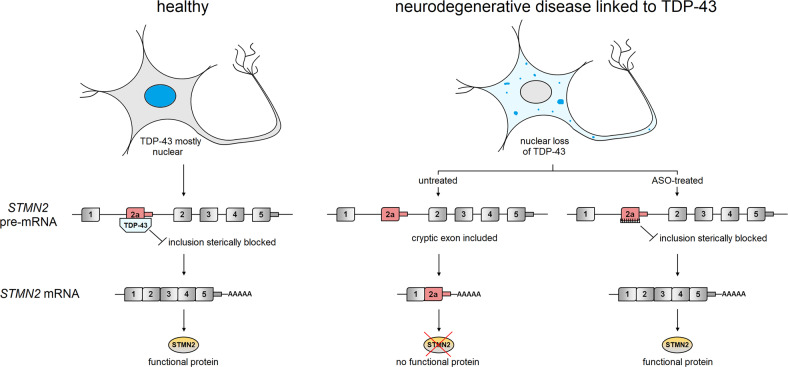
Summary of the findings by Baughn et al. In neurons of healthy individuals, TDP-43 is located predominantly in the nucleus and binds to *STMN2* pre-mRNA preventing inclusion of deleterious exon 2a. Nuclear loss of TDP-43 in vulnerable neurons occurs in a variety of neurodegenerative diseases, and inclusion of exon 2a in mature *STMN2* mRNA leads to the loss of STMN2 protein important for axon maintenance and regeneration. In *STMN2* pre-mRNA processing, TDP-43 acts as a steric block preventing access of the splicing machinery to exon 2a. This function of TDP-43 can be replaced, e.g., by antisense oligonucleotides (ASOs), and expression of STMN2 protein restored

Stathmin-2 (STMN2) is a microtubule-associated protein specifically expressed in neurons and required for axon outgrowth and maintenance, as well as for axon regeneration after injury in vitro. Loss or reduction of this protein in mice leads to progressive sensory and motor neuropathy resembling some crucial, but not all, features of ALS.^2,3^ TDP-43 is, at least in humans, essential for expression of functional full-length STMN2 protein. It binds to the pre-mRNA of *STMN2* and prevents inclusion of a cryptic exon (exon 2a) located in the first intron. Nuclear loss of TDP-43 in NDs induces exon 2a inclusion that introduces an in-frame stop-codon as well as a premature polyadenylation signal resulting in a severely truncated mRNA, and loss of functional STMN2 protein (Fig. 1). This aberrant mRNA, along with reduced *STMN2* full-length mRNA and protein, is detectable in *post-mortem* CNS tissue of ALS patients displaying TDP-43 pathology. Importantly, when considering that TDP-43 regulates many mRNAs, expression of STMN2 alone is sufficient to substantially rescue defects in axon regeneration of human iPSC-derived motoneurons after knockdown of TDP-43.^4^ These key findings suggest that restoration of *STMN2* expression may represent a promising therapeutic strategy for TDP-43-linked NDs, especially in sporadic cases. On the other hand, there are multiple additional pathogenic cascades triggered by the malfunction, mislocalization and/or aggregation of TDP-43 that contribute to the death of neurons in NDs, but are most likely not rescued by restoring *STMN2* expression alone. These cascades include defects in transcription, processing, turnover and axonal transport of multiple additional coding and non-coding RNAs, impairments of the DNA damage response, toxic effects of cytoplasmic TDP-43 on mitochondria, and the sequestration of proteins and RNAs into TDP-43 aggregates.^5^ Therefore, gaps in our knowledge of spatiotemporal events in TDP-43 proteinopathies currently prevent estimating benefits of rescuing *STMN2* expression for human patients.

Baughn et al. moved an important step forward toward a STMN2-based therapeutic approach for TDP-43 proteinopathies. First, they addressed molecular mechanisms of TDP-43-dependent cryptic exon 2a inclusion in *STMN2* mRNA. Previous data suggested that TDP-43 directly binds to a 24 bp GU-rich motif in exon 2a of the *STMN2* pre-mRNA. Replacing this motif with a 19 bp stem-loop forming sequence of the bacteriophage MS2 by genome editing of human neuroblastoma cell line SH-SY5Y led to deleterious exon 2a inclusion in *STMN2* mRNA. To rescue these defects, Baughn et al. expressed the MS2 coat protein (MCP) that binds to the introduced 19 bp stem-loop in *STMN2* pre-mRNA with high affinity. Here, both expression of MCP alone or fused to an inactive TDP-43 variant lacking the RNA-binding domains similarly prevented exon 2a inclusion and restored correct *STMN2* pre-mRNA splicing, supporting the hypothesis that TDP-43 functions as a simple steric block. This was further confirmed by introducing the human MS2-edited exon 2a including flanking regions into the first intron of the *Stmn2* gene of murine Neuro2a cells. In mice, exon 2a is not conserved and *Stmn2* pre-mRNA processing is not dependent on TDP-43. However, this humanization of mouse *Stmn2* induced similar processing defects as reported in the human SH-SY5Y cells, and could be rescued by expression of MCP. Additionally, in SH-SY5Y cells homozygously expressing the ALS-related N352S variant of TDP-43 and displaying the described defects of *STMN2* pre-mRNA processing, the authors show that not only MCP, but also the nuclease-dead CRISPR effector RfxCas13d (dCasRx) can compensate for the steric block function of TDP-43 when guided to the right position in exon 2a. Further genome editing of SH-SY5Y cells revealed that the 3’ splice acceptor site, but not the premature polyadenylation signal, of exon 2a is responsible for cryptic splicing after TDP-43 depletion. Taken together, these very elegant experiments, that were also controlled for possible effects on endogenous TDP-43 expression, leave little doubt that TDP-43 functions as a steric block for the cryptic 3’ splice acceptor site of deleterious exon 2a in *STMN2* pre-mRNA processing.^1^

Next, Baughn et al. developed an antisense-oligonucleotide (ASO)-based approach to compensate for TDP-43 function in *STMN2* pre-mRNA processing (Fig. 1). 250 ASOs that do not recruit RNase H and bind in and around exon 2a were screened for rescuing *STMN2* expression in SH-SY5Y cells homozygously expressing TDP-43 N352S. The binding sites of the five best-performing ASOs were located immediately up- or downstream of the binding sites for TDP-43 in exon 2a. Functionally, using iPSC-derived motoneurons, the authors show that phenotypes induced by knockdown of TDP-43, such as impaired axon regeneration and lysosome trafficking as well as abnormal increase of electron dense material within synapses, are substantially rescued when *STMN2* expression is restored with ASOs. Additionally, Baughn et al. provide evidence for the feasibility of their ASO-based treatment approach in vivo. In two slightly different mouse lines engineered for misprocessing of *Stmn2* pre-mRNA by heterozygously inserting MS2-edited exon 2a in intron 1 of the gene, intracerebral ventricular injection of ASOs was capable of rescuing *Stmn2* expression at the mRNA and protein level in cortex and spinal cord. Here, two administrations of a specific ASO restored *Stmn2* mRNA and protein expression from 50% to 75%, and from 25% to 80%, respectively, of wildtype mice.^1^

Besides these highly promising results, Baughn et al. additionally found that homozygous humanization of *Stmn2* in ALS model mice expressing the TDP-43 variant Q331K does not induce misprocessing of *Stmn2* pre-mRNA, or worsen phenotypes such as reduced grip strength. Considering that these mice do not show TDP-43 pathology, these results confirm the authors’ hypothesis that indeed nuclear loss of TDP-43 is required to induce misprocessing of *Stmn2* pre-mRNA.^1^ However, this finding emphasizes existence of additional TDP-43-dependent mechanisms beyond nuclear loss that contribute to NDs. Nonetheless, humanization of *Stmn2* in ALS model mice more closely resembling human TDP-43 neuropathology may represent an excellent approach to quantify the contribution of TDP-43 induced reduction of Stmn2 protein to the degenerative phenotype, and may simultaneously provide an ideal model to determine potential benefits of restoring *Stmn2* expression using ASOs.

In conclusion, while Baughn et al. report substantial progress in the development of a STMN2-based treatment strategy for TDP-43 proteinopathies, it remains to be determined if rescuing expression of a single downstream target of TDP-43 is sufficient to markedly delay disease progression in humans. Nevertheless, this approach is among the most promising for treating sporadic NDs linked to TDP-43, and is definitively worth being further followed-up.
